# Spatial and Temporal Relationships Between Roe and Red Deer in an Alpine Area

**DOI:** 10.1002/ece3.70777

**Published:** 2025-01-18

**Authors:** Valerio Donini, Luca Pedrotti, Francesco Ferretti, Elisa Iacona, Lucrezia Lorenzetti, Francesca Cozzi, Luca Corlatti

**Affiliations:** ^1^ Department of Life Science University of Siena Siena Italy; ^2^ Stelvio National Park Bormio Italy; ^3^ Sustainable Development and Protected Areas Service Stelvio National Park Cogolo di Pejo Autonomous Province of Trento Italy; ^4^ National Biodiversity Future Center Palermo Italy; ^5^ Chair of Wildlife Ecology and Management University of Freiburg Freiburg Germany

**Keywords:** activity patterns, camera traps, *Capreolus capreolus*, *Cervus elaphus*, competition, detection, occupancy

## Abstract

Interspecific interactions are important drivers of population dynamics and species distribution. These relationships can increase niche partitioning between sympatric species, which can differentiate space and time use or modify their feeding strategies. Roe deer *Capreolus capreolus* and red deer *Cervus elaphus* are two of the most widespread ungulate species in Europe and show spatial and dietary overlap. However, limited information is available on their interspecific relationships, especially in mountainous areas. In this study we used 5 years of camera trapping data collected in the Stelvio National Park (Central Italian Alps) to investigate spatial and temporal interactions between roe deer and red deer. Analyses were based on 2060 and 9030 roe deer and red deer detections, respectively, collected from July to September, from 2019 to 2023, using 50 camera traps randomly distributed over a 10,000‐ha study area. Spatial interactions were assessed by fitting a single‐season, single‐species occupancy model to calculate the probability of roe deer detection and occupancy as a function of relative red deer abundance and site‐specific environmental covariates. Temporal interactions were obtained by comparing the diel activity patterns of the two species. Results showed no significant effect of red deer relative abundance on the probability of presence of roe deer. Spatial analysis suggested a higher probability of roe deer presence in forested habitats, at lower elevations, and in areas with gentler slopes. Diel activity patterns of roe deer were consistent across sites with higher and lower red deer relative abundance, with moderate to high interspecific overlap, suggesting moderate temporal partitioning and no major support for temporal avoidance of the latter by the former. The high degree of overlap between the two species may be the result of area‐specific ecological conditions, such as the widespread distribution of red deer during the summer period, as well as of the adoption of strategies that favor coexistence.

## Graphical abstract

The roe deer and red deer are two species that are widely distributed in alpine environments. We used camera traps to investigate whether red deer relative abundance affects roe deer occupancy probability and whether the two species exhibit patterns of temporal segregation. The results suggested that the red deer had no effects on roe deer occupancy. However, a partial temporal segregation between the two species was observed.

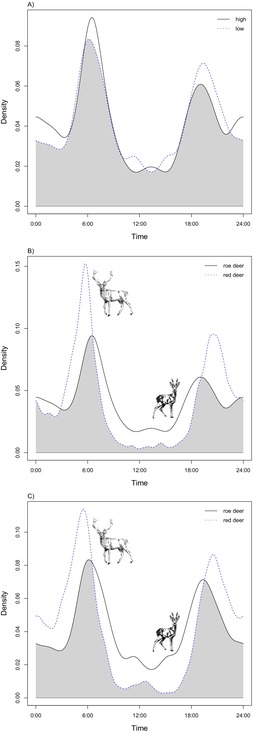

## 1 Introduction

A comprehensive understanding of the mechanisms underlying interspecific interactions is of paramount importance to ecological studies, as they are major drivers of population dynamics and species distribution (Schoener 1973). In general, five types of interactions have been described in animal communities: mutualism, commensalism, parasitism, predation, and competition (Krebs 1985). In mutualism and commensalism, at least one of the species benefits from the interaction without causing harm to the other. In contrast, the other interactions result in costs to one or more species (Krebs 1985). Interspecific competition can occur when sympatric species share the same limited resources, resulting in a reduction of growth, fecundity, and/or survival of at least one of the actors (De Boer and Prins 1990). Interspecific competition can occur through two main mechanisms: interference and resource exploitation. The former involves aggressive behavior that triggers avoidance responses in the inferior competitor (Schoener 1983; Holdridge, Cuellar‐Gempeler, and terHorst 2016). The latter refers to the reduction of resource availability by one species for the other (Lang and Benbow 2013). However, the nature of competitive interactions varies considerably depending on environmental and resource variability, and it is often difficult to distinguish the underlying mechanisms (Darmon et al. 2012).

Niche partitioning usually facilitates coexistence among sympatric species, ultimately leading to the evolution of behavioral and ecological adaptive strategies. These may include a differentiation in the use of space and time or in feeding strategies (Schoener 1974). The partitioning of space or time may prove an effective strategy for avoiding areas with high densities of the dominant species (Durant 1998) or for reducing temporal overlap to decrease the risk of competitive encounters (Carothers, Jaksić, and Jaksic 1984; Kronfeld‐Schor and Dayan 2003). The investigation of spatial and temporal relationships has typically been conducted separately, with the combination of these factors receiving comparatively little attention in the context of sympatric species (Lewis et al. 2015). This may be due to the inherent difficulties in studying interspecific interactions (Darmon et al. 2012), as these relationships may be contingent on a multitude of intrinsic and extrinsic factors (Zanni et al. 2021), including population density, resource availability, and the potential for shared predation risk (De Boer and Prins 1990).

The coexistence of species can be facilitated by landscape heterogeneity in both space and time, particularly when species differ in body size (De Boer and Prins 1990). Nevertheless, interspecific interactions may also occur between species of different body sizes (Richard et al. 2010). For example, the red deer *Cervus elaphus* is known to be a superior competitor to several other ungulate species, and it can affect the distribution and survival rate of smaller species such as the Alpine chamois *Rupicapra rupicapra rupicapra* (Corlatti et al. 2019; Kavčić et al. 2021) and the Apennine chamois *Rupicapra pyrenaica ornata* (Lovari et al. 2014; Ferretti et al. 2015). Conversely, the roe deer *Capreolus capreolus* is often considered a subordinate species, and previous studies suggested negative relationships with sympatric ungulates such as the muntjac *Muntiacus reevesi* (Hemami and Dolman 2005) and the fallow deer *Dama dama* (Focardi et al. 2006; Ferretti, Sforzi, and Lovari 2011). Furthermore, behavioral interactions have been reported between roe deer and sika deer *Cervus nippon* or fallow deer, with the latter preventing roe deer access to feeding areas (Danilkin 1996), also through direct aggression (Ferretti, Sforzi, and Lovari 2008; Ferretti, Sforzi, and Lovari 2011). On a temporal scale, previous studies suggested a high degree of overlap between the activity rhythms of roe deer and competing species such as wild boar *Sus scrofa* and fallow deer (Zanni et al. 2021). From the available literature it can be reasonably suggested that interspecific interactions between red deer and roe deer may occur, with the potential for negative effects on the latter. Nevertheless, there is a paucity of information regarding their spatial and temporal interactions.

Roe deer and red deer are widespread in Europe, and their distribution overlaps in many ecosystems (Apollonio, Andersen, and Putman 2010). The red deer is considered an intermediate feeder, and it can exploit both high‐quality food and poor‐quality food (Hofmann 1989). On the contrary, the roe deer is a concentrated selector, thus feeding on more digestible forage (Hofmann 1989). Because of this, a potential for diet overlap can occur, consequently leading to a potential for competitive interactions. Although these two species are both native to European temperate ecosystems, under certain conditions negative effects on roe deer populations caused by high red deer densities have been found throughout Europe (Scotland: Latham, Staines, and Gorman 1997; France: Richard et al. 2010; Poland: Borkowski et al. 2021). Previous studies have shown that higher red deer densities may reduce the body mass of roe deer fawns, with implications for population dynamics (Richard et al. 2010). Furthermore, evidence of a negative effect of red deer density on roe deer spatial distribution has been found in Portugal, suggesting the vulnerability of the latter to the former (Torres et al. 2012). However, no information exists for Alpine environments, and specifically, little is known about interspecific interactions between these two deer species on a spatial and temporal scale.

The present study aims to investigate the spatial and temporal interactions between the red deer and the roe deer in an Alpine protected area. The potential effects of the relative abundance of red deer on key aspects of time and space use of roe deer were analyzed through occupancy models using data from 5 years of intensive summer monitoring with camera traps. Based on the available literature, we hypothesize that roe deer occupancy should be influenced by the relative abundance of red deer and that a diel temporal segregation between the two species during the summer period is to be expected. We anticipate that a spatial and temporal partitioning should occur between the two species, due to (i) a negative effect of the relative abundance of red deer on the occupancy of roe deer and (ii) a reduced interspecific temporal overlap in sites that are highly used by the red deer, in comparison with sites with a low relative abundance of red deer.

## 2 Materials and Methods

### 2.1 Study Area

**FIGURE 1:**
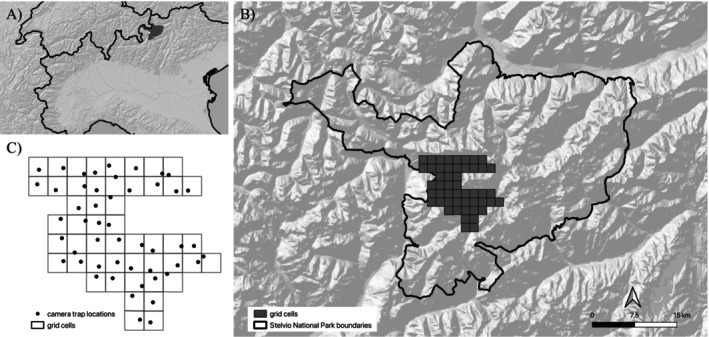
Location of the Stelvio National Park (grey polygon) in the central Italian Alps (A) and the boundaries of the study area within the Park (B). Panel (C) illustrates the arrangement of grid cells and camera trap locations, which were positioned according to a random tessellation approach.

The study area was located in the Stelvio National Park (Central Italian Alps, 46°27′ N, 10°25′ E; Figure 1A). The area extends over approximately 10,000 ha and is characterized by an Alpine climate, with yearly mean temperature ranging between −2.8°C in winter and 15.7°C in summer (Corlatti, Gugiatti, and Pedrotti 2016). Altitudes range from 1500 m to 2700 m a.s.l. (above sea level), and vegetation is mainly composed of spruce *Picea abies* , larch *Larix decidua* , and stone pine *Pinus cembra* forests at lower elevations, and Alpine grassland composed of *Carex curvula*, *Festuca helleri*, *Selseria varia*, and *Carex firma* above the tree line. Shrub vegetation is characterized by the presence of rhododendron *Rhododendron ferrugineum* and *Rhododendron hirsutum*, and mountain pine, *Pinus mugo*, while at the highest elevations (above 2700 m a.s.l.) landscape is characterized by rock cover and glaciers. In addition to roe deer and red deer, the study area hosts stable populations of Alpine chamois and ibex *Capra ibex* . The presence of large carnivores such as wolves *Canis lupus* , brown bears *Ursus arctos* , or Eurasian *lynx Lynx lynx* was rare. The Stelvio National Park is home to one of the highest densities of red deer in Europe (Mattioli et al. 2022), while the density of roe deer is low. Within the Park, roe deer and red deer populations were monitored on a yearly basis using spring spotlight counts. Every year in April and May, the same routes were driven during the night for at least three consecutive nights, and animals were counted using spotlights. This method reliably tracks changes in red deer abundance and, within our study area, it allows to approximate absolute population size after applying a rate of underestimation of 0.52 to the raw counts (Corlatti, Gugiatti, and Pedrotti 2016). During the surveys, roe deer sightings were also noted down. Although this approach has not been validated for the local roe deer population, its standardized use may provide a minimum number of individuals useful for monitoring purposes within the study area. From 2009 to 2023, the mean raw count for red deer within the study area was 550 individuals, with a standard deviation of ±200. During the same period, the mean raw count for roe deer was 13 individuals (SD ± 6). During the winter period (December–February), a limited culling program is carried out on about 25% of the study area, with approximately 150 deer hunted by personnel authorized by the Stelvio National Park.

### 2.2 Data Collection

The spatio‐temporal interactions between roe deer and red deer were investigated using camera traps. From 2019 to 2023, 50 camera traps were deployed, and sampling was conducted between May and October of each year. The location of the camera traps was defined using a random tessellation approach (Stevens and Olsen 2003; cf. Moeller et al. 2023): the study area was initially divided into 50 grid cells of 1.5 km^2^, and a random point was selected within each of them to represent the location of the camera trap (Figure 1C). This approach should guarantee unbiased sampling of all environmental characteristics and ensure the homogeneous coverage of the study area. The positioning of the camera traps remained consistent throughout the duration of the study. The cameras were positioned at an approximate height of 50 cm above the ground, facing north wherever feasible, to minimize the potential for sun‐induced triggers. The cameras were programmed to take pictures on a 24‐h basis throughout the entire study period. They were then checked on a regular basis, at intervals of between 20 and 25 days, to download the images and replace the batteries when necessary. Four distinct infrared camera trap models with a nocturnal black flash were utilized: the Doerr Snapshot Mini Black 5.0, the Cuddeback C123, the Bushnell Trophy Cam HD Aggressor, and the Comitel Guard 1. A total of 120 “Bushnell,” 64 “Comitel,” 47 “Cuddeback” and 19 “Dorr” camera traps were used during the study period. The images downloaded from the camera traps were processed using the Timelapse2 program (Greenberg, Godin, and Whittington 2019). Each image was classified according to the species detected, the number of individuals, and their sex and age. We considered each image of the species taken at least 5 min apart from the next/previous image as a single independent event (following Henrich et al. 2022). If an image or series of images taken within this 5‐min interval included more than one individual, each clearly recognizable individual was treated as a single independent event (based on sex and age characteristics). To accommodate data in an occupancy framework, for each year (primary sampling occasion), the roe deer data were organized into nine secondary sampling occasions between 1 July and 30 September, each occasion including 10 days of sampling. The length of the primary sampling occasion was chosen to minimize the possibility of migration and immigration (Rota et al. 2009). For each secondary sampling occasion, the presence (coded as 1) and absence (coded as 0) of roe deer were recorded.

### 2.3 Detection Covariates

To model the probability of detecting roe deer, data were collected on the environmental characteristics of the area surrounding each camera trap. First, the amount of undergrowth cover in the vicinity of the camera trap was quantified. It was assumed that this index could account for any imperfect detection resulting from the presence of shrub cover, trees, or stones in front of the camera traps. A black and white rectangular sheet (42 × 60 cm, comprising 16 black and white squares) was positioned in front of the camera trap at a distance of 13 m (based on field tests), at two heights (0 and 130 cm) and in three directions (45° to the left, 45° to the right, and in front). The number of squares that were not visible was then counted, and the index was calculated as a percentage. The amount of forest cover above each camera trap was also assessed. This variable, hereafter named ‘canopy cover,’ should account for the different probability of detecting an animal in open or closed environments. The ability of the camera trap to detect an animal passing in front of it can be affected by factors such as sunlight and wind in open areas or shadows in the forest (Hofmeester et al. 2019). Accordingly, a photograph of the sky was taken at a distance of 13 m from the camera trap, at a height of 130 cm above the ground. This image was then analyzed using ImageJ software (version 1.52k) to ascertain the percentage of the area covered by tree crowns. Finally, the number of operational days for each camera trap was calculated over a 10‐day period to account for the specific camera trapping effort at each site.

### 2.4 Occupancy Predictor and Covariates

To investigate whether red deer relative abundance exerted an influence on the probability of roe deer occupancy, the trapping rate of the former was incorporated as the target predictor. In particular, for each camera trap site and year of sampling, the number of independent red deer events was calculated by summing up the events (i.e., number of red deer) and dividing by the number of days the camera trap was active. Although multi‐species occupancy models could have been employed, the widespread distribution of red deer indicated that red deer capture rates would be a more appropriate metric. The red deer index was subsequently employed as a continuous independent variable. Moreover, to control for potential drivers of roe deer occupancy, environmental features were extracted from a vegetation map provided by the Lombardy Region (DUSAF, Agricultural and Forestry Land Use Destination). In particular, the proportion of coniferous and shrubby vegetation was determined within a 250‐m radius around each camera trap location. The preliminary analyses indicated that the size of the buffer was representative of the environmental characteristics of the entire study area. In addition, to test whether the probability of roe deer occupancy was influenced by the presence of water and by the proximity to grassland, the minimum distance in meters from each camera trap site to each of these features was calculated. The median values of elevation, exposure, and slope were extracted from a digital terrain model (DTM) map within each buffer. The exposure variable was transformed into an index of northness, and the slope variable was transformed into degrees. The northness value was calculated as the cosine of the exposure value, with a range of 1 (representing a completely north‐exposed slope) to −1 (representing a completely south‐exposed slope). To assess the anthropogenic impact, a human presence index was obtained using a raster map of resolution 20 × 20 m. The map was derived from Strava, a mobile application used by hikers, runners, and cyclists, which offers heatmaps of trail and road usage. These heatmaps can be used to extract a Cumulative Outdoor activity Index, ranging from 0 to 100 (Corradini et al. 2021). As this index considers the recreational effect of human presence, a second variable was calculated to account for the effect of the proximity to humans on the probability of roe deer occupancy. Specifically, the minimum distance in meters to human infrastructure (i.e., roads and settlements) from each camera trap site was calculated.

### 2.5 Occupancy Modeling

Although roe deer data were collected over several years, given the limited sample size (*n* = 50 camera trap locations per year) and the sparseness of roe deer data, roe deer occupancy was assessed using a single‐season occupancy model (MacKenzie et al. 2002, 2017). To accommodate data for this type of model, we stacked the roe deer capture history by year, following Fuller, Linden, and Royle (2016) and Linden et al. (2017). As a result, the dataset consisted of one row per year for each camera‐trapping site (i.e., 50 by 5 rows). Furthermore, males and females were combined to reduce issues of sampling variability due to limited sample size. Year, camera trap model, canopy cover, cover in front of the camera, and working days were used as detection covariates. Red deer trapping rate was the target predictor, while year, red deer trapping rate, forest cover, shrub cover, distance from water sources, from grasslands and human settlements, elevation, northness, eastness, and slope were used as occupancy covariates. All continuous variables were *z*‐transformed prior to analysis. The model was fitted using the “unmarked” package version 1.4.1; (Fiske and Chandler 2011) in R statistical software version 4.4.0 (R Core Team 2024) using RStudio version 2024.04.17 (Posit Team 2024). The models for detection (ρ) and occupancy (ψ) were fitted with a logit‐link function as follows:

$$\begin{array}{c} \text{logit}\;\left(\rho_{\mathrm{ij}}\right)=\alpha_{0}+\alpha_{2020}+\alpha_{2021}+\alpha_{2022}+\alpha_{2023}+\alpha_{\mathrm{CT}\;{\text{model}}_{i}}\\ +\alpha_{\text{canopy}\;{\text{cover}}_{i}}+\alpha_{\text{cover in front of}\;{\text{the camera}}_{i}}+\alpha_{\text{working}\;{\text{days}}_{i}} \end{array}$$

$$\begin{array}{c} \text{logit}\;\left(\psi_{i}\right)=\beta_{0}+\beta_{2020}+\beta_{2021}+\beta_{2022}+\beta_{2023}+\beta_{\mathrm{red}\;\text{deer}\;{\text{trapping rate}}_{i}}\\ +\beta_{\text{forest}\;{\text{cover}}_{i}}+\beta_{\text{shrub}\;{\text{cover}}_{i}}+\beta_{\text{distance}\;{\text{water}}_{i}}+\beta_{\text{distance}\;\text{human}\;{\text{settlements}}_{i}}\\ +\beta_{\text{distance}\;{\text{grasslands}}_{i}}+\beta_{{\text{human presence}}_{i}}+\beta_{{\text{elevation}}_{i}}+\beta_{{\text{northness}}_{i}}+\beta_{{\text{slope}}_{i}} \end{array}$$

$\rho_{\mathrm{ij}}$ equation refers to the probability of roe deer being detected at *i*th site and *j*th sampling occasion, accounting for year effect, site‐specific covariates, and survey‐specific covariates. $\psi_{i}$ equation refers to the probability of roe deer to occupy a *i*th site, accounting for the effect of year, red deer trapping rate, and site‐specific environmental features. To test collinearity, the VIF (Variance Inflation Factor) values were calculated using the “vif” function of the “unmarked” package. All variables had VIF < 3. To test the goodness of fit of the models, three different tests were used: sum of squared errors, Pearson's Chi‐squared, and Freeman‐Tukey Chi‐squared. These tests were performed using the “parboot” function in the “unmarked” package using 1000 replications.

### 2.6 Temporal Analysis

The temporal association between roe deer and red deer at camera trap sites was investigated using the “activity” (version 1.3.4; Rowcliffe et al. 2014) and “overlap” (version 0.3.9; Meredith, Ridout, and Campbell 2024) R packages. Two distinct approaches were employed. Firstly, we investigated whether roe deer showed different patterns of diel activity related to the intensity of red deer relative abundance. In particular, the objective was to ascertain whether the higher or lower relative abundance of red deer at camera‐trapping sites was associated with different patterns of roe deer activity. The sites were classified as “higher” or “lower” in terms of red deer relative abundance, based on whether the red deer trapping rate was above or below the mean value. Subsequently, the activity pattern of the roe deer was examined within each of the two groups, and the degree of overlap between the roe deer patterns was quantified. Subsequently, we investigated whether the activity patterns of red deer and roe deer exhibited similarities under varying conditions of red deer relative abundance. In contrast to the previous approach, we also considered the red deer activity rhythms and investigated whether the roe deer would respond to it under conditions of “higher” and “lower” red deer relative abundance. by investigating the degree of overlap. A non‐parametric kernel density approach was employed to estimate the activity patterns of the deer species. The time of each camera trap detection was initially transformed into radians using the formula: (((hours × 3600 + min × 60))/π)/43,200. To evaluate the degree of overlap between the pairwise activity curves, the coefficient of overlap (Δ) was calculated using the “overlapEst” function (R package ‘overlap,’ Meredith, Ridout, and Campbell 2024). This coefficient quantifies the extent of overlap between two kernel density estimates, considering the shared area under both curves. The Δ coefficient returns a value that ranges from 0 (indicating no overlap) to 1 (indicating complete overlap; Ridout and Linkie 2009). In particular, the Δ_4_ coefficient was employed, which is typically advised for substantial sample sizes (i.e., over 75 camera records; Meredith and Ridout 2014). Δ_4_ overlapping values were considered as “moderate” between 0.50 and 0.75, as “high” between 0.75 and 0.90, and as “very high” above 0.90 (Monterroso, Alves, and Ferreras 2013). For each overlap index, the 95% confidence intervals were calculated using a bootstrap with 1000 resamples, following Meredith and Ridout (2014). The Watson‐Wheeler (*W*) test was calculated with the R package “circular” (version 0.5‐0; Agostinelli and Lund 2023) for each pairwise temporal analysis to test for differences between the circular distributions, i.e., the activity curves (Batschelet 1981). The density curves were plotted using the overlapPlot function (R package “overlap”, Meredith, Ridout, and Campbell 2024).

## 3 Results

Overall, a total of 2069 and 9030 independent events of roe deer and red deer, respectively were generated from camera traps. Specifically, the average number of independent roe deer events per year was 414 (SD = ±50), while the yearly number of red deer events was 1806 (SD = ±133). A total of 812 and 1257 independent roe deer events were extracted from the camera trapping sites at high and low red deer relative densities, respectively. In addition, 712 roe deer independent events were females, 853 males, and 504 undefined (which includes both kids and unidentified adults). The camera traps were active for a total of 21,490 days, and each was active for an average of 86 days out of the 92 days available (SD = ±1.7).

Model diagnostics did not show any major violation of model assumption. The goodness of fit tests returned for the sum of squared errors a numeric vector of statistic (*t*_0_) of 333 and a *p*‐value of 0.820, for Pearson's Chi‐squared a numeric vector of statistic (*t*_0_) of 1179 and a *p*‐value of 0.999, and for Freeman‐Tukey Chi‐squared a numeric vector of statistic (*t*_0_) of 430 and a *p*‐value of 0.721.

### 3.1 Detection Pattern

**FIGURE 2:**
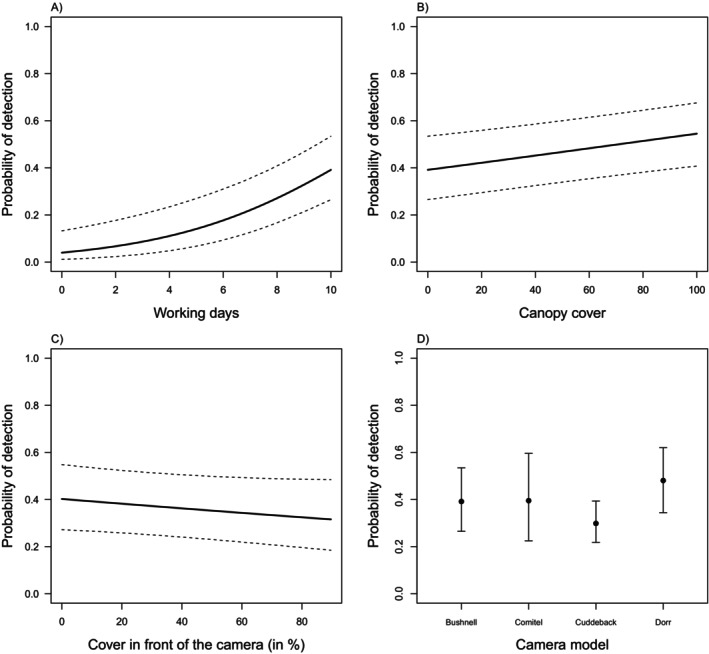
Marginal probability of camera trap detection as a function of the number of working days (A), the canopy cover (B), the percentage of cover in front of the camera traps, (C) and the model of the camera traps (D).

Model results suggested that roe deer detection probability increased as the number of working days increased (Table 1, Figure 2A). The probability of detection was also positively influenced by the canopy cover, suggesting a higher tendency of roe deer to be detected when in forested areas (Table 1, Figure 2B). Although not statistically significant, the probability of roe deer detection decreased as the coverage in front of the camera trap increased (Table 1, Figure 2C). Furthermore, the model of the camera trap affected the probability of detecting the roe deer, and the results suggest that the ‘Cuddeback’ model had a lower detection probability than the others, and the ‘Dorr’ had the highest probability (Table 1, Figure 2D).

**TABLE 1:** Parameter estimates of the occupancy and detection model built to investigate roe deer occupancy probability within the study area located in the Stelvio National Park between 2019 and 2023.

	Parameter	Estimate	SE	*p*
Detection	(Intercept)	−0.430	0.280	0.125
	**Year [2020 vs. 2019]**	**0.594**	**0.284**	**0.037**
	**Year [2021 vs. 2019]**	**0.774**	**0.283**	**0.006**
	Year [2022 vs. 2019]	0.048	0.317	0.879
	Year [2023 vs. 2019]	0.460	0.427	0.281
	**Working days**	**0.512**	**0.113**	**< 0.001**
	Model [Comitel vs. Bushnell]	0.012	0.311	0.968
	Model [Cuddeback vs. Bushnell]	−0.419	0.231	0.069
	Model [Dorr vs. Bushnell]	0.349	0.387	0.367
	**Canopy cover**	**0.208**	**0.062**	**0.001**
	Cover in front of the camera	−0.083	0.062	0.186
Occupancy	(Intercept)	0.048	0.441	0.914
	Year [2020 vs. 2019]	−0.130	0.578	0.823
	Year [2021 vs. 2019]	0.164	0.573	0.775
	Year [2022 vs. 2019]	0.522	0.582	0.370
	Year [2023 vs. 2019]	0.759	0.573	0.185
	**Conifers cover**	**0.130**	**0.366**	**< 0.001**
	Red deer trapping rate	0.010	0.191	0.958
	Shrub cover	0.444	0.233	0.057
	Human presence	−0.196	0.393	0.618
	Northness	−0.173	0.186	0.350
	**Slope**	**−0.825**	**0.233**	**< 0.001**
	Elevation	−0.707	0.483	0.143
	Distance to water sources	−0.171	0.229	0.457
	Distance to human settlements	−0.483	0.264	0.067
	Distance to grasslands	−0.308	0.283	0.276

*Note:* The table reports the parameter estimates (on the logit scale), the standard error (SE), and *p*‐values for detection (on top) and occupancy (on bottom) probability over 5 years (from 2019 to 2023) within the study area in the Stelvio National Park. Statistically significant parameters are in bold.

### 3.2 Occupancy Pattern

**FIGURE 3:**
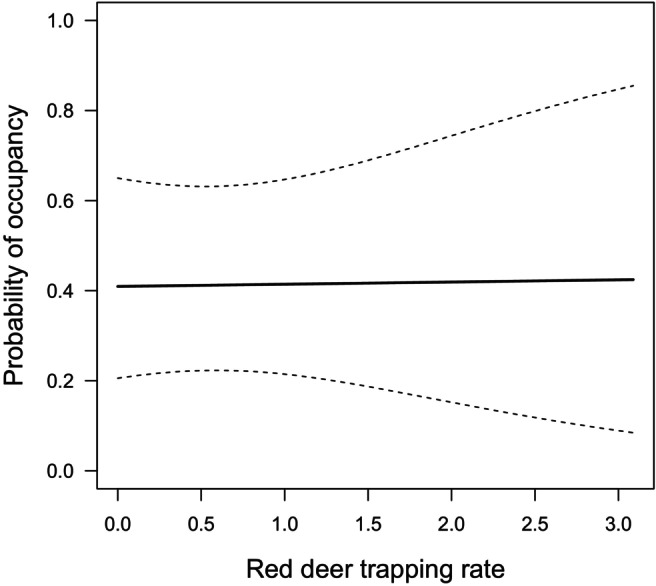
Marginal probability of camera trap occupancy as a function of red deer trapping rate.

**FIGURE 4:**
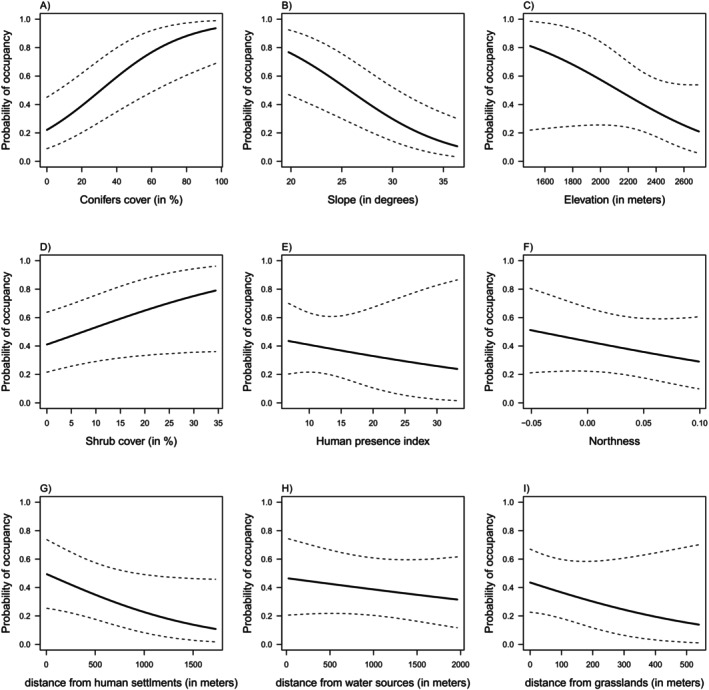
Marginal probability of camera trap occupancy as a function of the conifers cover (A), slope (B), elevation (C), shrub cover (D), human presence (E), northness (F), distance from human settlements (G), distance from water sources (H), and distance from grasslands (I).

Model results suggested no evidence for an effect of the relative abundance of red deer on the probability of occupancy of roe deer (Table 1, Figure 3). The occupancy probability was influenced by the percentage of conifer cover and slope (Table 1, Figure 4A–C). Results show that roe deer preferred forested habitats (Table 1, Figure 4A), with gentler slopes (Table 1, Figure 4B). Although not significant, there was slight support for roe deer occupancy being positively associated with areas with a higher percentage of shrub cover (Table 1, Figure 4D) and closer to human settlements (Table 1, Figure 4G). No significant support was found for elevation (Table 1, Figure 4C), human presence (Table 1, Figure 4E), northness (Table 1, Figure 4F), distance to water sources (Table 1, Figure 4H) and distance to grasslands (Table 1, Figure 4I).

### 3.3 Temporal Analysis

**FIGURE 5:**
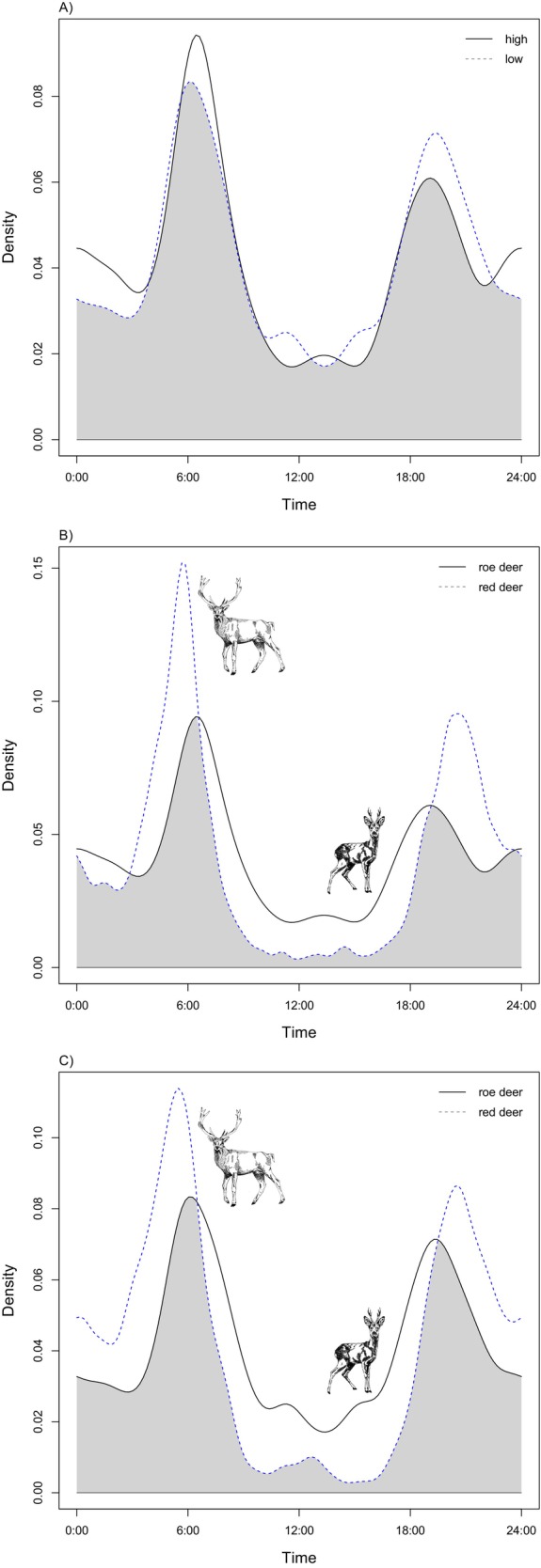
Panel (A) shows roe deer diel activity overlap between sites with lower (dashed line) and higher (straight line) red deer trapping rates. Panel (B) and (C) show roe deer (straight line) and red deer (dashed line) diel activity overlap at sites at higher (B) and lower (C) red deer trapping rates. The gray shaded area represents the temporal pairwise overlap. Drawings by Chiara Giari ©.

Overall, the two species showed a strong bimodal pattern, suggesting two peaks of activity at dusk and dawn (Figure 5). The overlap (Δ_4_) between roe deer activity patterns in sites with higher and lower red deer trapping rates was 0.94 (95% CI: 0.89–0.95; Figure 5A), and the Watson‐Wheeler test value (*W*) of 0.99 returned a *p*‐value of 0.61. At sites where red deer relative abundance was higher, roe deer and red deer temporal overlap was 0.74 (95% CI: 0.69–0.75; Figure 5B), and the Watson‐Wheeler test value (*W*) was 123.05 with *p*‐value < 0.01, while at lower red density sites the overlap was 0.76 (95% CI: 0.74–0.79; Figure 5C) and value *W* of 246.33 with *p*‐value < 0.01.

## 4 Discussion

Our results suggest that the relative abundance of red deer did not affect the probability of occupancy of roe deer, which was mainly influenced by the vegetation composition and orographic features. On a temporal scale, roe deer showed no significant differences in diel activity between sites with low and high red deer occurrence. However, some evidence for a different temporal pattern between the two species was found.

In contrast with our predictions, the relative abundance of red deer did not have a negative impact on the probability of roe deer occupancy, indicating that the two species co‐occurred in space. Additionally, sex‐specific analyses were carried out for male and female roe deer to investigate whether red deer trapping rate may affect the spatial behavior of each sex. However, no red deer effect was found for females, while a weak positive association was found for males (Material S1: Data S1). The role of red deer abundance in shaping the spatial distribution of roe deer has been sparsely examined in previous studies. In Portugal, Torres et al. (2012) observed that even at relatively low red deer densities (2.3–4.7 individuals per 100 ha), red deer can exert a negative influence on the spatial use of roe deer. Similar conclusions were reached in Slowinski National Park (Poland), where the analysis of pellet group counts suggested a negative impact of red deer density on roe deer spatial distribution (Borkowski et al. 2021). At a larger spatial scale, a negative relationship was identified between the densities of these two deer species across 20 forested areas in Scotland, thereby supporting the hypothesis that red deer density has a negative effect on roe deer abundance (Latham, Staines, and Gorman 1996, 1997). The findings of these studies have been interpreted as the result of resource exploitation. An adult red deer is four to five times larger in body size than a roe deer. When the former occupies the same areas at considerably higher densities than the latter, as evidenced by studies such as Borkowski et al. (2021) and the present study, the potential for resource exploitation is expected to be higher. Consequently, the presence of red deer can impact the foraging opportunities of roe deer, which in turn affects their fitness and spatial distribution (Latham 1999). In our study area, the red deer naïve occupancy models yielded a probability of occupancy of 0.94 and a probability of detection of 0.61 within the same study period. This suggests that the red deer was extensively distributed throughout the study area, and it is conceivable that roe deer had no other places to occupy, other than those already occupied by red deer.

In contexts where two species co‐occur in close spatial proximity and share the same resources, temporal segregation may also occur. This phenomenon is manifested in the inferior competitor concentrating its activities during different periods of the day with respect to those used by the superior competitor. Examples of time partitioning are common in ecological studies (Di Bitetti et al. 2010), including those of ungulate species (Šprem et al. 2015; Kavčić et al. 2021). The activity peaks of roe deer occurred at dawn and dusk, a bimodal pattern that has been documented across diverse environmental conditions (Cederlund 1989; Chapman et al. 1993; Pagon et al. 2013; Rossa, Lovari, and Ferretti 2021). The results suggested no modification in the temporal behavior of roe deer in relation to local levels of red deer activity. Similarly, the temporal overlap between roe deer and red deer was found to be consistently moderate to high across sites with high and low red deer trapping rates (in accordance with the definition proposed by Monterroso, Alves, and Ferreras 2013). However, the activity rhythms of roe deer and red deer showed significant differences between the two species. These discrepancies are evident in both the activity peaks observed at dawn and dusk, with red deer exhibiting more pronounced peaks than roe deer, and in the daytime activity patterns, which appear to be more prominent in roe deer. However, the observed differences appear to be consistent across sites with higher versus lower red deer relative abundance and also across both roe deer sexes (Material S2: Data S1). These results may indicate that temporal avoidance strategies were not employed by the roe deer to reduce its overlap with the red deer. On the other hand, we cannot rule out the possibility that the widespread presence of red deer and their potential impact on roe deer in the past may have independently altered the activity rhythms of roe deer at both high and low densities. Similar results have been observed in a Mediterranean area, where roe deer activity patterns overlapped with those of fallow deer during the summer period (Zanni et al. 2021).

A limitation of this study is the restricted temporal range of sampling, which was confined to the summer season. Negative interactions are often most prevalent during the winter months (Borkowski et al. 2021), when animals are compelled to occupy smaller areas at the bottom of the valleys, leading to increased competition for food resources. During the summer months, particularly in mountainous regions, animal populations are more evenly distributed. Moreover, red deer occur at high densities and are widespread in our study area, whereas roe deer are relatively rare. A negative effect of increasing red deer densities on roe deer abundance may have occurred before this study bagan when red deer exhibited a marked expansion within the study area (cf. Latham, Staines, and Gorman 1997; Torres et al. 2012; Borkowski et al. 2021). We suggest that the marked differences in the density of the two species may also be the result of such a long‐term interaction process. If so, the current patterns of spatial and temporal relationships between roe deer and red deer may be the result of previous interactions that could not be detected during our investigation. In turn, we suggest that historical interactions may have led to the current low densities of roe deer, which would be forced to share space and time with the red deer.

Our results suggested that roe deer occupancy was mainly influenced by the landscape characteristics. We found strong evidence for an effect of conifer forest and slope, suggesting a greater use of closed habitats and flat areas in summer. These results align with those of other studies, possibly indicating that canopy closure may favor thermal regulation and protection against extreme weather conditions (Mysterud and Østbye 1973; Vospernik and Reimoser 2008; Mancinelli et al. 2015). Our findings also showed a weak preference for areas with more shrub cover. Roe deer are concentrate selectors (Hofmann 1989), which may explain their use of habitats with higher food quality, such as those represented by shrub regeneration. We also found a weak negative association with distance to human settlements, suggesting that roe deer are more likely to occupy areas close to human infrastructures. On the contrary, no effects were found for the human presence index. This discrepancy may be due to the fact that human presence refers to the recreational activities of people in the area, while distance to infrastructure refers to the physical presence of roads or buildings. In our study area, anthropized areas are located at lower altitudes with gentler slopes and are close to wooded areas, i.e., suitable habitats for the roe deer, which may explain our findings. Furthermore, there was only a little evidence of a negative relationship with distance from grasslands and elevation, suggesting that roe deer, which are often associated with ecotonal habitats (Apollonio, Andersen, and Putman 2010; Lovari, Serrao, and Mori 2017), may prefer sites close to meadows and at lower elevations. As reported in other studies conducted in the Alps (Mancinelli et al. 2015), we did not find support for a strong effect of exposure on roe deer occupancy probability, but only a tendency towards southern‐exposed slopes. In addition, no evidence for an effect of distance to water sources was found, although the presence of water, especially during the dry season, has been documented as an important factor for roe deer (Tellería and Virgós 1997; Wallach et al. 2007).

Overall, red deer trapping rate did not play a significant role in affecting roe deer spatial and temporal distribution. The presence of roe deer seemed to be more influenced by favorable feeding conditions and by the presence of shelters (presence of forest and shrubs at low altitudes) than by the presence of red deer, which is ubiquitous throughout the study area. A comparison with study areas exhibiting disparate deer densities may yield further insight. A more detailed analysis of interspecific dynamics between these species is therefore required, for example, through diet analysis or thorough examination of potential effects on physiological traits. This study may provide the basis for future ecological and management considerations. For example, further investigations are needed on the effects of the culling of red deer in the study area and whether this has an impact on the roe deer population through density‐dependent mechanisms. Moreover, the recent recolonization of wolves, *Canis lupus* , in the Italian Alps (Fabbri et al. 2007; Marucco et al. 2018) may impact these relationships. An investigation of wolf effects in a multi‐prey system may yield different results with respect to the interaction between roe and red deer.

## Author Contributions

**Valerio Donini:** data curation (lead), formal analysis (lead), writing – original draft (lead). **Luca Pedrotti:** conceptualization (lead), supervision (lead), writing – review and editing (equal). **Francesco Ferretti:** conceptualization (lead), supervision (lead), writing – review and editing (equal). **Elisa Iacona:** data curation (equal), writing – review and editing (supporting). **Lucrezia Lorenzetti:** data curation (equal), writing – review and editing (equal). **Francesca Cozzi:** data curation (equal), writing – review and editing (supporting). **Luca Corlatti:** conceptualization (lead), formal analysis (lead), methodology (equal), supervision (lead), writing – original draft (equal), writing – review and editing (equal).

## Conflicts of Interest

The authors declare no conflicts of interest.

## Supporting information


Data S1.

## Data Availability

Data and Script are available at: https://doi.org/10.5281/zenodo.13869276.
