# Supplementary material for: Spatial and Temporal Relationships Between Roe and Red Deer in an Alpine Area
Source: Ecol Evol. 2025 Jan 18;15(1):e70777. doi: 10.1002/ece3.70777 (PMC11748455; doi:10.1002/ece3.70777)
Supplement: Supplementary file 1 — Data S1. [file ECE3-15-e70777-s001.docx]

**Supplementary material for manuscript: “Spatial and temporal relationships between roe and red deer in an Alpine area”**

**S1: sex-specific difference in roe deer probability of occupancy in relation to red deer trapping rate**

Here we present the spatial analysis conducted separately for female and male roe deer camera trapping data, from 2019 to 2023 within the Stelvio National Park.

*Females*

The table reports the results of the occupancy model built to investigate female roe deer probability of detection and occupancy in relation to red deer trapping rate and environmental characteristics. For each parameter is reported the estimate, standard error and p-value. Statistically significant parameters are in bold.

|  | Parameter | Estimate | SE | *p*-value |
| --- | --- | --- | --- | --- |
| *Detection females* |  |  |  |  |
|  | **(Intercept)** | **-0.943** | **0.413** | **0.022** |
|  | Year [2020 vs 2019] | 0.289 | 0.403 | 0.473 |
|  | Year [2021 vs 2019] | 0.324 | 0.389 | 0.405 |
|  | Year [2022 vs 2019] | 0.059 | 0.427 | 0.890 |
|  | Year [2023 vs 2019] | 0.376 | 0.527 | 0.476 |
|  | **Working days** | **0.627** | **0.204** | **0.002** |
|  | Model [Comitel vs Bushnell] | -0.002 | 0.342 | 0.996 |
|  | Model [Cuddeback vs Bushnell] | -0.326 | 0.313 | 0.297 |
|  | Model [Dorr vs Bushnell] | 0.585 | 0.506 | 0.248 |
|  | Canopy cover | 0.133 | 0.074 | 0.073 |
|  | Cover in front of the camera | -0.067 | 0.078 | 0.387 |
| *Occupancy females* |  |  |  |  |
|  | (Intercept) | -0,632 | 0,431 | 0,142 |
|  | Year [2020 vs 2019] | 0,059 | 0,585 | 0,920 |
|  | Year [2021 vs 2019] | 0,522 | 0,591 | 0,378 |
|  | Year [2022 vs 2019] | 0,916 | 0,602 | 0,128 |
|  | Year [2023 vs 2019] | 0,869 | 0,576 | 0,131 |
|  | **Conifers cover** | **0,150** | **0,332** | **<0.001** |
|  | Red deer trapping rate | -0,088 | 0,197 | 0,655 |
|  | **Shrub cover** | **0,718** | **0,251** | **0,004** |
|  | Human presence | -0,226 | 0,255 | 0,374 |
|  | Northness | 0,219 | 0,194 | 0,260 |
|  | **Slope** | -0,385 | 0,219 | 0,079 |
|  | Elevation | -0,387 | 0,372 | 0,299 |
|  | Distance to water | -0,074 | 0,202 | 0,714 |
|  | Distance to human settlements | -0,317 | 0,246 | 0,198 |
|  | Distance to grasslands | -0,471 | 0,243 | 0,052 |

The Figure shows the marginal probability of camera trap occupancy for female model as a function of red deer trapping rate.

The figure shows the marginal probability of camera trap occupancy for female model as a function of the conifers cover (Panel A), slope (Panel B), elevation (Panel C), shrub cover (Panel D), human presence (Panel E), northness (Panel F) distance from human settlements (Panel G), distance from water sources (Panel H) and distance from grasslands (Panel I).

*Males*

The table reports the results of the occupancy model built to investigate male roe deer probability of detection and occupancy in relation to red deer trapping rate and environmental characteristics. For each parameter is reported the estimate, standard error and p-value. Statistically significant parameters are in bold.

|  | Parameter | Estimate | SE | *p*-value |
| --- | --- | --- | --- | --- |
| *Detection males* |  |  |  |  |
|  | (Intercept) | -0,498 | 0,468 | 0,287 |
|  | **Year [2020 vs 2019]** | 0,019 | 0,452 | 0,967 |
|  | **Year [2021 vs 2019]** | 0,210 | 0,446 | 0,638 |
|  | Year [2022 vs 2019] | -0,361 | 0,488 | 0,459 |
|  | Year [2023 vs 2019] | 0,134 | 0,582 | 0,818 |
|  | **Working days** | **0,466** | **0,144** | **0,001** |
|  | Model [Comitel vs Bushnell] | 0,034 | 0,354 | 0,924 |
|  | **Model [Cuddeback vs Bushnell]** | **-0,766** | **0,359** | **0,033** |
|  | Model [Dorr vs Bushnell] | 0,148 | 0,556 | 0,791 |
|  | Canopy cover | 0,093 | 0,075 | 0,215 |
|  | Cover in front of the camera | -0,034 | 0,073 | 0,639 |
| *Occupancy males* |  |  |  |  |
|  | (Intercept) | -0,114 | 0,422 | 0,007 |
|  | Year [2020 vs 2019] | 0,646 | 0,557 | 0,246 |
|  | Year [2021 vs 2019] | 0,790 | 0,553 | 0,153 |
|  | **Year [2022 vs 2019]** | **0,118** | **0,567** | **0,038** |
|  | Year [2023 vs 2019] | 0,105 | 0,548 | 0,056 |
|  | **Conifers cover** | **0,101** | **0,319** | **0,002** |
|  | Red deer trapping rate | 0,371 | 0,193 | 0,054 |
|  | **Shrub cover** | **0,502** | **0,212** | **0,018** |
|  | Human presence | 0,260 | 0,254 | 0,307 |
|  | Northness | 0,256 | 0,178 | 0,150 |
|  | Slope | -0,275 | 0,205 | 0,179 |
|  | Elevation | -0,445 | 0,366 | 0,224 |
|  | Distance to water | 0,373 | 0,207 | 0,072 |
|  | **Distance to human settlements** | **-0,738** | **0,313** | **0,018** |
|  | Distance to grasslands | -0,336 | 0,238 | 0,159 |

The Figure shows the marginal probability of camera trap occupancy for male model as a function of red deer trapping rate.

The figure shows the marginal probability of camera trap occupancy for male model as a function of the conifers cover (Panel A), slope (Panel B), elevation (Panel C), shrub cover (Panel D), human presence (Panel E), northness (Panel F) distance from human settlements (Panel G), distance from water sources (Panel H) and distance from grasslands (Panel I).

**S2: sex-specific difference in roe deer activity patterns in relation to red deer**

Here we present the temporal analysis conducted using female and male roe deer camera trapping data.

Diel activity overlap between female roe deer (straight line) and red deer (dashed line). The grey shaded area represents the temporal pairwise overlap. Dhat4 overlapping value is 0.721.

Diel activity overlap between male roe deer (straight line) and red deer (dashed line). The grey shaded area represents the temporal pairwise overlap. Dhat4 overlapping value is 0.730.
